# Measuring quantities using oscillators and pulse generators

**DOI:** 10.1007/s12064-012-0153-4

**Published:** 2012-05-25

**Authors:** Maciej Komosinski

**Affiliations:** Institute of Computing Science, Poznan University of Technology, Piotrowo 2, 60-965 Poznan, Poland

**Keywords:** Clock–counter model, Spike, Neuron, Periodic, Time, Scalar

## Abstract

This article presents properties of the clock–counter model with a periodic generator employed as the source of regularly emitted pulses. The pacemaker and accumulator mechanisms are often considered in research in neurobiology and cognitive science: neurons or their groups serve as oscillators, and the number of spikes emitted while a stimulus lasts becomes an estimate of the length of the stimulus. The article integrates three approaches: a theoretical model to present the general concept, a working implementation of this model to perform intensive simulation experiments, and the analytical description of the behavior of the model. Oscillators that exhibit some degree of regularity have been compared to the Poisson ones, and the corresponding probability distributions have been presented that describe the number of pulses accumulated over time. Several continuous and discrete interpulse distributions have been investigated, and the influence of generator parameters on the possible outcomes of the measurement have been described. Particular attention has been paid to the relationship between measurement variability and the mean number of pulses observed. Issues concerning practical realizations of periodic generators: discrete time, dependence of the generator start time on the stimulus, and relation to Weber’s law have been discussed as well.

## Introduction

The motivation of this research is to investigate properties of a clock–counter (or a pacemaker–accumulator) architecture that is used to measure continuous or discrete quantities, and employs a periodic generator as a source of pulses. Such generators can be constructed—both in biological systems and in engineering—from simple oscillators, and can be used to transform magnitudes of “analog” phenomena (time, frequency, brightness, temperature, force, and pressure) to their discrete estimates. This is equivalent to a digital measurement of a quantity.

**Fig. 1:**
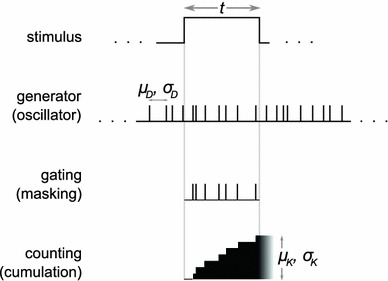
The considered method for measurement of quantities. The generator is often called a clock or a pacemaker. Note that the terms *masking* and *gating* have different meanings in neurobiology, psychology, and technology. This method is used in the well-known clock–counter models (Grondi 2010; Grondi 2001; Ivry 2008; Ulrich et al. 2006; Bueti 2011)

It is interesting to note that in engineering and electronics several approaches are known to convert an analog signal to its discrete representation. Employing an oscillator (or a “clock”) to estimate the magnitude of some quantity is one of these approaches—one that is particularly suitable for biological systems. In nature, the oscillator can take the form of a neural circuit, where groups of neurons generate oscillatory activity with modulated frequency (Matell 2004; Gerstner 1999). This activity serves as a spike generator; spikes are then accumulated into a discrete estimate that reflects the actual amount of the perceived phenomenon, as shown in Fig. 1. An attractive property of this architecture is that the generator and the counter can be separated from the stimulus; the stimulus is only used for gating and does not directly affect other components of the measuring system.

This article discusses properties of such architecture, specifically, the precision of measurements that can be achieved when using periodic generators. The relationship between the magnitude of the stimulus and the uncertainty of its estimate is important, as this relationship is known to obey specific laws in animals and humans (Grondin 2001; Gibbon 1977; Gescheider 1997); still, there are various controversies on this subject (Wearden 2008; Bizo 2006; Lewis 2009; Rammsayer 2001; Rammsayer 2000; Kang et al. 2010). Similar models have been studied earlier as the models of perception and making judgments regarding stimuli magnitudes; see for example (Gibbon 1992; Gibbon et al. 1984; Rammsayer 2001), cf. also (Wearden et al. 2007; Wearden 1999; Buhusi 2005; Ulrich et al. 2006; Ivry 2008).

To illustrate characteristics of this model and the influence of generator parameters on the possible outcomes of the measurement, specialized computer simulations of the pulse generation and counting processes have been developed and performed. For precision, some of the presented results are averages from as many as 10^10^ simulation runs.

## The model and its properties

The following setting is considered: an oscillator is available that can generate pulses with known mean interpulse (interspike) interval length μ_*D*_ and variance σ_*D*_^2^. The number of pulses *K* counted within time *t* is investigated; in particular, the average number of pulses μ_*K*_ and its variance σ_*K*_^2^.

Note that this is different from the setting where it is the number of pulses, *k*, that is assumed to be fixed (σ_*K*_^2^ = 0), and the time *T* is the random variable, its mean and variance being investigated. This would correspond to these situations when exactly *k* pulses must occur and one is interested in the time it takes for the pulses to occur (Killeen 1987; Getty 1976). In this work, another situation is considered: it is the time *t* that elapses, and one is interested in the number of pulses *K* that could have occurred within this time (the number of pulses *K* is an estimate of time *t*). Both situations are related; but, since time is continuous and the number of pulses is discrete, they are not equivalent.

The considered architecture corresponds to a stochastic process known as a counting process, with pulses being counted events (also called arrivals). Since time intervals between pulses are assumed to be independent and identically distributed, this counting process is a renewal process (Smith 1958; Cox 1962). Indeed, each pulse is a renewal: once it occurs, the interpulse cycle repeats.

## Characteristics of the generator

The time between spikes varies according to some distribution *D* with mean μ_*D*_ and variance σ_*D*_^2^. In particular, the following distributions have been tested in simulation:

- Exponential distribution, Exp(λ). Since μ_*D*_ is the mean interval length, $\lambda=1/\mu_D.$
- Normal distribution, $$\mathcal{N}(\mu_D,{\sigma_D}^2). $$
- Uniform continuous distribution,* U*($\mu_D-\sigma_D\sqrt 3,\mu_D+\sigma_D\sqrt 3$).
- Two-point distribution, a spike generated with probability $1/2$ at μ_*D*_ − σ_*D*_ or μ_*D*_ + σ_*D*_.

The exponential distribution of interval length corresponds to the case where the pulse generation is a Poissonian process. The normal, uniform, and two-point distributions require that a generator (an oscillator) is more complex and can generate pulses with some degree of regularity, albeit not necessarily perfectly periodically (hence σ_*D*_^2^ > 0).

## The memoryless generator

For a Poisson process, where the events occur continuously and independently at a constant average rate λ, intervals between events follow the exponential distribution, Exp(λ), and $\lambda=\frac{1}{\mu_D}.$ The mean number of pulses occurring in time *t* depends linearly on $t, \mu_K=\frac{t}{\mu_D}=\lambda t.$ The variance of the number of the pulses in time *t* and its relations to the mean number of pulses are as follows:

$$ \begin{aligned} &{\sigma_K}^2= \mu_K \\ &\frac{{\sigma_K}^2}{\mu_K}= 1 \\ &\frac{\sigma_K}{\mu_K}=\frac{1}{\sqrt{\mu_K}} \end{aligned} $$

## Periodic generators: the triggered and the non-triggered case

For periodic generators, two cases are considered. In one case, it is assumed that pulse generation and observation are related: either pulse generation is somehow triggered by the fact of the observation, or the observation begins in response to the generated pulse. In the other case, these two processes are independent. The former case where the first pulse is not counted is the ordinary renewal process, while the latter one is the equilibrium renewal process (Cox 1962).

**Fig. 2:**
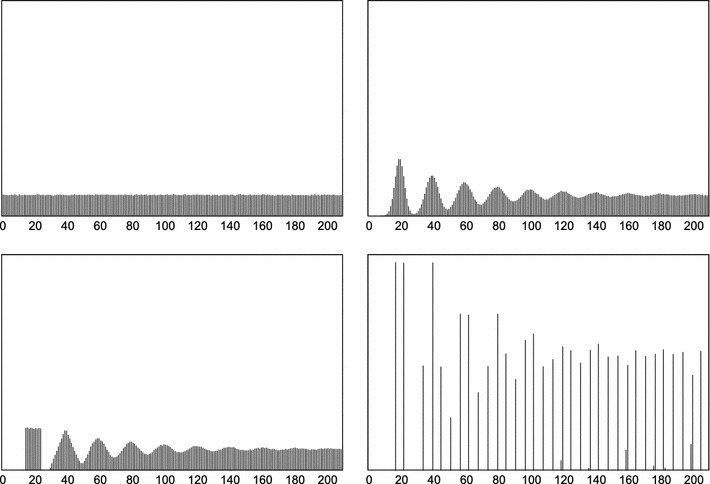
The relative number of pulses (*vertical axis*) occurring in time (*horizontal axis*) in the triggered setup. The first generated pulse occurs at time moment −1 (i.e., one time unit before the observation period starts). Mean interpulse time is μ_*D*_ = 20. From* top to bottom, left to right*: exponential ($\lambda=\frac{1}{20}$), normal, uniform, and two-point distribution of interpulse time *D*. For normal, uniform, and two-point distributions, ${\sigma_D}^2=\frac{25}{3}, \sigma_D\approx 2.9.$

Fig. 2 shows the average number of pulses that occur in time moment *t*, assuming that the first pulse was generated at time −1. In the long term, for generators with interpulse interval distribution that has a continuous component, the probability of observing a pulse in a specific moment does not depend on the particular distribution of *D*.

In the non-triggered case, the generator is not influenced by the “start time” effect illustrated in Fig. 2—it is independent from the stimulus. In other words, all start times of the generator are equally probable. Differences between triggered and non-triggered generators are further discussed in sections “Periodic generators” and “Independent (non-triggered) versus triggered generators”.

The independence of pulse generation and observation guarantees that for any distribution of interpulse intervals, the mean number of pulses

1 $$ \mu_K=\frac{t}{\mu_D} $$

which ensures that there is no systematic error introduced by the generator, and on average, *K* reflects the length of the stimulus, *t* (which may in turn correspond to the magnitude of the measured, primary stimulus, were it not time). This intuitive relation is an important property known as mean accuracy (Wearden 2003).

Since the oscillator is characterized by μ_*D*_ > 0 and σ_*D*_^2^, and these two values are assumed to be invariable, a parameter

2 $$ c=\left(\frac{\sigma_D}{\mu_D}\right)^2 $$

(squared coefficient of variation) is introduced that describes an oscillator and is constant for a particular oscillator.

## Periodic generators

**Fig. 3:**
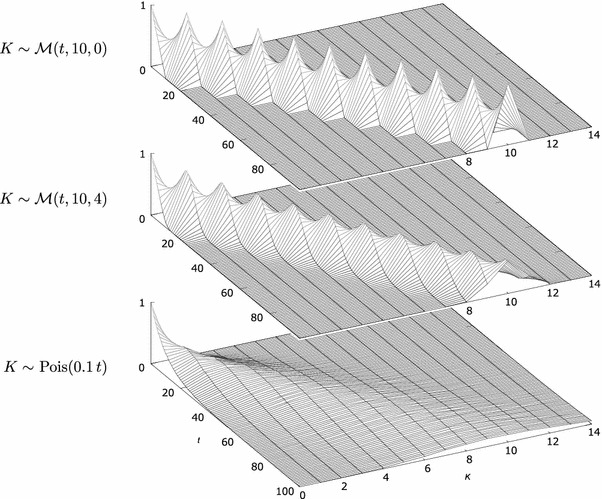
Probability (*vertical axis*) of observing *K* pulses in time *t* in the non-triggered setup. The *K* axis is discrete, and the lines are only guides for the eye. Mean interpulse time is μ_*D*_ = 10.* Top*: a perfectly periodic oscillator, σ_*D*_^2^ = 0.* Middle*: interpulse time is normally distributed, σ_*D*_^2^ = 4.* Bottom*: interpulse time is exponentially distributed, $\lambda=\frac{1} {\mu_{D}}.$

The random variable *K* is the number of pulses *k* ($k=0,1,2,\ldots$) in time *t*, given the pulse generator characterized by μ_*D*_ and σ_*D*_^2^. *K* has a discrete distribution denoted here as $\mathcal{M}(t,\mu_D,{\sigma_D}^2)$ and illustrated in Fig. 3. This distribution will be characterized below to show how it arises from *D* and to provide a link between distributions enumerated in “Characteristics of the generator” section and the outcomes shown in Figs. 2 and 3. For a more extensive analytical treatment of the renewal processes, refer to (Cox 1962).

Let us first consider the triggered case, when the first counted pulse in time interval of length *t* always occurs immediately once the interval begins. The following pulses are generated independently and the mean interval between consecutive pulses has a length of μ_*D*_. Therefore, the probability that *k* pulses fit the interval of length *t* is described by the following cumulative distribution function^1^ of *K*:

3 $$ \begin{aligned} F_K^{\rm triggered}(k;t,\mu_D,{\sigma_D}^2)&={ P}(K \leq k)\\ &=1-F_{\rm normal}(t;\mu=k\cdot\mu_D,\sigma^2=k\cdot{\sigma_D}^2)= 1-\Upphi\left(\frac{t-k\cdot\mu_D}{\sqrt{k\cdot{\sigma_D}^2}}\right) \end{aligned} $$

**Fig. 4:**
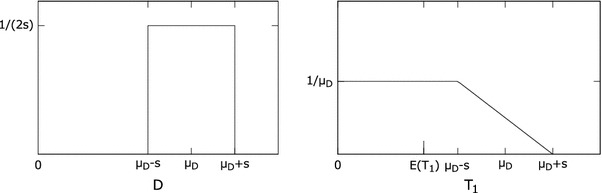
Probability density function of the time of first pulse, *T*_1_ (*right graph*) for uniformly distributed interpulse time *D* (*left graph*)

In the non-triggered setup, one has to take into account the fact that the observation period *t* occurs anywhere in the sequence of pulses. Therefore, the first pulse that occurs in time window *t* needs to be considered specially. The probability of the time moment when the first pulse occurs, *T*_1_, depends on *D*, and consequently should be determined specifically for each *D*. As an example, *T*_1_ is determined here for the uniformly distributed interpulse time. Let μ_*D*_ be the mean interpulse time, and *s*—half the width of variability of interpulse time, μ_*D*_ ≥ *s* and *s* ≥ 0, as shown in Fig. 4.

The mean and variance of time of the first pulse occurring, *T*_1_, are therefore

$$ \begin{aligned} & {\text{E}}(T_1) =\int_0^{\mu_D-s}\frac{t}{\mu_{D}} {\text d}t + \int_{\mu_D-s}^{\mu_D+s}t\frac{\mu_D+s-t}{2s \mu_D} {\text d}t = \frac{\mu_D}{2} +\frac{{s}^{2}}{6\mu_D}\\ &  \hbox{Var}(T_1)=\int_0^{\mu_D-s}\frac{(t-\text{E}(T_1))^2}{\mu_D} \hbox{d}t +\int_{\mu_D-s}^{\mu_D+s} \frac{(t-\text{E}(T_1))^2 \cdot(\mu_D+s-t)}{2s \mu_D} \hbox{d}t\\ &\qquad\,=\frac{{\mu_D}^{2}}{12} -\frac{{s}^{4}}{36{\mu_D}^{2}}+\frac{{s}^{2}}{6} \end{aligned} $$

and since for uniform distribution that has been considered $s=\sigma_D \sqrt{3},$ the first pulse has the following expected time and variance:

$$ \begin{aligned} &\text{E}(T_1)=\frac{\mu_D}{2} +\frac{{\sigma_D}^2}{2\mu_D}\\&\quad{\text{Var}}(T_1)=\frac{{\mu_D}^{2}}{12} -\frac{{\sigma_D}^{4}}{4 {\mu_D}^{2}} + \frac{{\sigma_D}^{2}}{2}\end{aligned} $$

The offset of the first pulse has to be included in time period of length *t* along with the *k* intervals between pulses. Note that *T*_1_ has a different distribution than *D* so adding their means and variances together will not represent the pulse generation process perfectly accurately; this will be illustrated in “The *r* component of variance σ_*K*_^2^” section. Considering probability of time of the first pulse, *T*_1_, yields the cumulative distribution function of *K* for the non-triggered case to be

4 $$ \begin{aligned} F_K(k;t,\mu_D,{\sigma_D}^2)&=1-F_{\rm normal}(t;\mu=k\cdot\mu_D+\text{E}(T_1),\sigma^2=k\cdot{\sigma_D}^2+\hbox{Var}(T_1))\\ &=1-\Upphi\left(\frac{t-k\cdot\mu_D-\text{E}(T_1)} {\sqrt{k\cdot{\sigma_D}^2+\hbox{Var}(T_1)}}\right) \end{aligned} $$

and one can note that the triggered setup is a special case of the non-triggered one, where E(*T*_1_) = 0 and Var(*T*_1_) = 0.

Probability mass function

$$ f_K(k)={P}(K=k)=F_K(k)-F_K(k-1) $$

and *F*_*K*_(*k*) = 0 for *k* < 0. The mean value of *K*

$$ \mu_K=\sum_{k=0}^\infty k\cdot f_K(k) $$

which, for the non-triggered case, follows Eq. 1.

The remainder of this section discusses the behavior of the variance of *K*.

## A perfectly periodic oscillator

Consider a perfect, periodic, non-triggered generator with σ_*D*_ = 0. Since there elapses exactly time μ_*D*_ between each pair of generated pulses, the variance of *K* for time *t* will only depend on the relation between *t* and μ_*D*_. It will specifically depend on the remainder of *t* and μ_*D*_ and will therefore be periodic in *t* with a period of μ_*D*_. This is illustrated in the top plot in Fig. 3.

**Fig. 5:**
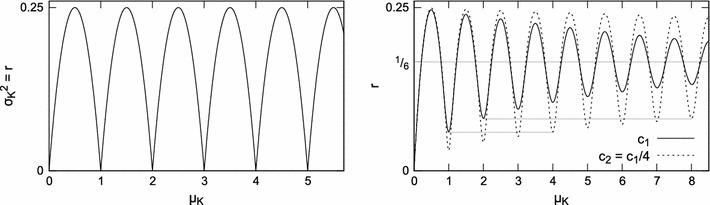
Oscillations of σ_*K*_^2^. Note that the horizontal axis shows the mean number of pulses, not *t*—see Eq. 1. *Left*: a perfectly periodic generator (*c* = 0).* Right*: comparison of convergence of the component *r* of σ_*K*_^2^ for two generators characterized by *c*_1_ and *c*_2_ (see Eq. 2). For small *c*, the rate of convergence is approximately proportional to *c*: since *c*_1_ is four times bigger than *c*_2_, the convergence of *r* for the first generator is four times faster than for the second one, cf. Fig. 8

If *t* is a multiple of μ_*D*_, then the number of observed pulses *K* is always μ_*K*_ (Eq. 1); *K* does not depend on the generator offset (or start time) and thus σ_*K*_^2^ = 0. On the other hand, for μ_*D*_ twice as long as *t*, the number of pulses *K* that occur in time *t* varies: in fifty percent of cases one pulse occurs, and in the remaining cases no pulse is found in time *t*. The variance of *K* contributed by this situation will therefore be maximum. Between these two extreme cases, the values of σ_*K*_^2^ will be intermediate depending on the remainder of the (integer quotient) division of *t* by μ_*D*_, as illustrated in Fig. 5, left.

When performing *x* experiments, the variance of *K* is

$$ {\sigma_K}^2=\frac{\sum_{i=1}^{x}{k_i^2} -\left(\sum_{i=1}^{x}{k_i}\right)^2/x}{x-1} $$

where *k*_*i*_ is the number of pulses observed in the *i*-th experiment. Since a generator with σ_*D*_^2^ = 0 is considered, the number of pulses counted in the intervals of length *t* may only differ by one, i.e., there may be *k* or *k* + 1 pulses, where $k=\lfloor \frac{t}{\mu_{D}}\rfloor.$ Let us assume that among *x* experiments, in *y* ≤ *x* experiments *k* pulses were observed, and in the remaining *x* − *y* experiments, *k* + 1 pulses occurred. Therefore, the variance

$$ {\sigma_K}^2=\frac{y\cdot k^2+(x-y)\cdot (k+1)^2-\left(y\cdot k+(x-y)\cdot (k+1)\right)^2/x}{x-1}=\frac{y(x-y)}{x(x-1)} $$

and it does not depend on *k*. The variance is zero for *y* = *x* or *y* = 0. For a large number of experiments ($$x\rightarrow\infty$$), the variance is maximum (0.25) for $$y=x/2. $$ For a small number of experiments *x*, the maximum σ_*K*_^2^ is $$\frac{x}{4(x-1)}$$ for even *x* and $$\frac{x+1}{4x}$$ for odd *x*.

Since the variance of *K* is periodic in *t* with a period of μ_*D*_, for each period of length μ_*D*_, *y* changes from *x* to 0. The end of each period (*y* = 0) is the beginning of the next one (*k* increases by 1 and *y* = *x*), therefore the mean variance of *K* for all *t* is

5 $$ \text{E}({\sigma_K}^2) = \frac{\sum\limits_{y=1}^x \frac{y(x-y)}{x(x-1)}}{x}=\frac{1}{6}+\frac{1}{6x} $$

which approaches $1/6$ as the number of experiments *x* grows.

### A non-perfectly periodic oscillator

For oscillators with σ_*D*_^2^ > 0, for short times *t*, the behavior is similar to the perfect σ_*D*_^2^ = 0 oscillator as the influence of the oscillator variance σ_*D*_^2^ on the variance of *K* is small. As *t* grows, the effect of randomness of consecutive pulse intervals cumulates and thus the number of different values *K* may take in each experiment increases (it is not just two values, *k* and *k* + 1, as in the σ_*D*_^2^ = 0 case).

The variance of the number of pulses observed in time *t* can be described as a sum of two components:

$$ {\sigma_K}^2=t\cdot\frac{{\sigma_D}^2}{{\mu_D}^3} + r_1(t,\mu_D,{\sigma_D}^2) $$

Considering Eqs. 1 and 2 yields

6 $$ {\sigma_K}^2=\mu_K\cdot c + r_2(t,\mu_K,c) $$

The first component causes the variance to grow linearly with *t* (Rammsayer 2001). The second component, *r* (of which *r*_1_ and *r*_2_ are just two alternative parametrizations), is the result of the regularity of the generator and the interplay between *t* and μ_*D*_ discussed in “A perfectly periodic oscillator” section. The *r* component constitutes σ_*K*_^2^ for the perfectly periodic generators; for *c* > 0 and non-triggered generators with non-skewed *D*, this component oscillates around $\frac{c^2}{2}+\frac{1}{6}$ (Cox 1962). For continuous *D*, the *r* component will converge while *t* grows; for small *c*, the convergence is faster for less regular oscillators (i.e., with higher *c*), as illustrated in the right graph in Fig. 5, the bottom left graph in Fig. 7 and in Fig. 8, left.

**Fig. 6:**
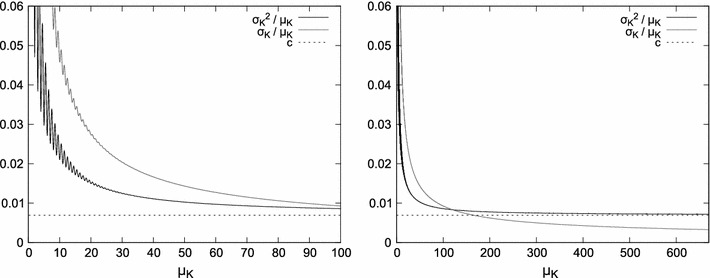
Variance-to-mean ratio and the coefficient of variation for a periodic generator with $\mu_D=12, {\sigma_D}^2=1, c=1/144\approx 0.007.$ The* left plot* shows an enlarged fragment of the* right plot*

**Fig. 7:**
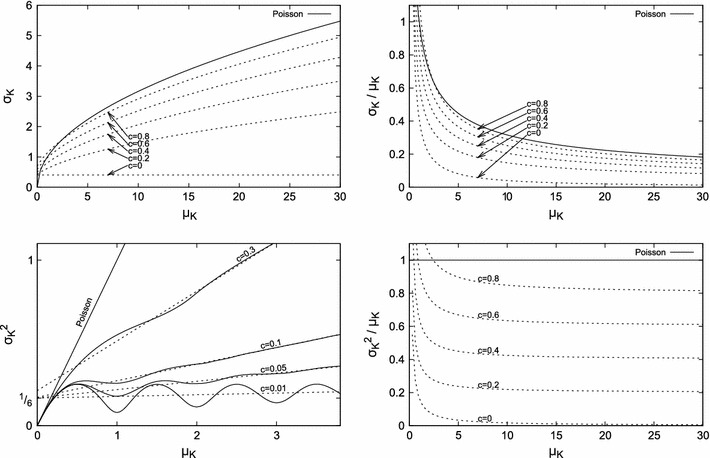
Comparison of Poissonian and periodic generators for varying *c*: four relations presented often in literature. All graphs show variance approximated by Eq. 7 (i.e., without oscillations of the component *r*). The* bottom left* graph additionally shows the actual variance (Eq. 6) as* solid lines*

**Fig. 8:**
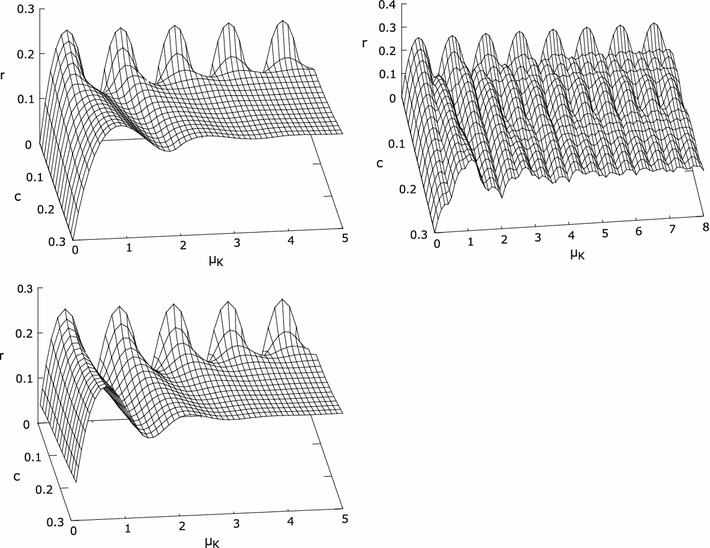
Comparison of oscillations of the *r* component of σ_*K*_^2^ for non-triggered generators with continuous uniform *D* (*top left plot*) and two-point *D* (*top right plot*), and a range of *c*. The* bottom plot* is based on Eq. 4. Note the shift in the locations of the extrema for increasing *c*

The *c* parameter is close to zero for highly regular oscillators; the μ_*D*_ ≥ *s* inequality that has been assumed for the uniformly distributed *D,* shown in Fig. 4, insures that $c\leq1/3.$ For uniformly distributed *D*, this condition guarantees that time that passes between consecutive pulses is nonnegative. Other distributions of *D* that yield periodic behavior of the generator may be characterized by higher values of *c* while still providing non-negative lengths of all interpulse intervals. An example is the two-point distribution defined in “Characteristics of the generator” section with σ_*D*_ ≤ μ_*D*_ and thus *c* ≤ 1.

## Asymptotic behavior of σ_*K*_^2^

For a large number of experiments with the non-triggered generator and non-skewed *D*, variance of the number of pulses observed in time *t* can be approximated and simplified from Eq. 6 to

7 $$ {\sigma_K}^2 \approx \mu_K\cdot c+\frac{c^2}{2}+\frac{1}{6} $$

Therefore, the variance-to-mean ratio

8 $$ \frac{{\sigma_K}^2}{\mu_K} \approx c+\frac{3 c^2+1}{6 \mu_K} $$

and the coefficient of variation

9 $$ \frac{\sigma_K}{\mu_K} \approx \frac{\sqrt{\mu_K\cdot c+\frac{c^2}{2}+\frac{1}{6}}}{\mu_K} $$

As μ_*K*_ grows to infinity (which is equivalent to *t* approaching infinity and an oscillator with finite μ_*D*_),

10 $$ \lim_{\mu_K \to \infty}{\sigma_K}^2 = \infty $$

11 $$ \lim_{\mu_K \to \infty}\frac{{\sigma_K}^2}{\mu_K}=c $$

12 $$ \lim_{\mu_K \to \infty}\frac{\sigma_K}{\mu_K}=0 $$

The limiting behavior of the coefficients in Eqs. 8 and 9 is important because it allows to distinguish between Poissonian, regular, and scalar (i.e., constant, non-zero coefficient of variation) models (Gibbon 1977), as discussed in “Relation to Weber’s law” section. Note that depending on the characteristics of the oscillator, *c*, the specific requirements of some experiment, and the available precision of measurements, the time *t* needed for subjective stabilization of the coefficient of variation, and the variance-to-mean ratio may vary, and may be considered “short” (i.e., not infinite as the equations above show), as illustrated in Fig. 6.

Comparing the variance of *K* for the Poisson generator against periodic generators, the difference is primarily caused by the *c* coefficient as shown in Fig. 7. To minimize σ_*K*_^2^, it is generally desirable to have *c* as small as possible (ideally, zero)—for *c*≈ 0, the value of σ_*K*_^2^ grows very slowly with *t*, yet its oscillations persist. On the other hand, in biological or biologically inspired systems, *c* may be much higher and the regularity of the oscillator much lower (or variable), thus making pulse generation more similar to the Poisson process that is often assumed in studies of the nervous system (Sejnowski 1999; Gibbon 1992; Rammsayer 2001), cf. (Kang et al. 2010).

## The *r* component of variance σ_*K*_^2^

The character of the oscillations of the *r* component around the base value depends on the interplay between probability distributions of consecutive pulses *D*, and—in case of the non-triggered generator—on *T*_1_. The oscillations decay with time for continuous distributions of *D*.

Characterizing *D* and *T*_1_ in terms of their means and variances suffices to describe asymptotic behavior of μ_*K*_ and σ_*K*_^2^ (Eq. 4), but more information about *D* and *T*_1_ is required to describe oscillations of *r* around the base value. Fig. 8 illustrates behaviors of these oscillations for the continuous uniform distribution and for the discrete two-point distribution of *D*. The *c* parameter varies from 0 to 0.3.

While continuous interpulse time distributions result in fading oscillations because probability distributions of consecutive intervals can interact and add, the discrete distribution of *D* produces a complex quasi-periodic landscape. Depending on the delay between pulses for the two-point *D* and the value of *c*, the pattern of oscillations varies. In particular, for discrete *D* the pattern will depend on the remainders of sums of delays between pulses and μ_*D*_. Note that in biology such discrete, extremely reproducible interpulse time distributions are unlikely to occur, and due to inherent inaccuracies of the substrate of the oscillator, the oscillations would eventually die out.

## Realizations of periodic generators

This section discusses in more detail three issues that concern practical implementations and existing realizations of periodic generators.

## Continuous versus discrete time

In experiments concerning time—which constitute a large part of experiments performed in biological and cognitive sciences—time is measured with a limited precision, using some kind of an external, discrete clock. This does not conflict with analyses presented in this article; here, time *t* is regarded as continuous, yet this concerns the internal time of the generator or oscillator, and not the external measurements of time performed while observing the behavior of the generator.

A different situation takes place when the architecture of the oscillator itself employs the concept of discrete time. This can result from some topologies of neural networks—one example is a helper periodic oscillator that feeds its pulses to the main oscillator. Discrete time is often encountered in simulations of biological processes and in technology, where it is implemented as a discrete variable (hence the notion of “time steps”). This is also convenient in settings where the generator is embedded in a network of units working synchronously (Adamatzky and Komosinski 2009; Komosinski and Adamatzky 2009), i.e., the network is not event-driven. Considerations presented here generally hold for such discrete-time settings as long as time steps are small enough; however, care must be taken to accurately estimate variances. This is obviously required when time steps are large and the difference between characteristics of the discrete and the continuous becomes apparent (e.g., for a discrete quasi-normally distributed pulse generator).

If generator time is considered discrete, the Poisson process of emitting pulses can be modeled by the memoryless Bernoulli process, where the time between pulses follows the geometric distribution, and the number *K* of pulses generated with probability *p* in time *t* is described by the binominal distribution, *B*(*t*, *p*). Since $p\,=\,1/ \mu_D$ and $\mu_K\,=\,p\cdot t,$

$$ \begin{aligned} {\sigma_K}^2\,=\,& p(1-p)\cdot t\,=\,\mu_K (1-p) \\ \frac{{\sigma_K}^2}{\mu_K}\,=\,& 1-p \\ \frac{\sigma_K}{\mu_K}\,=\,&\sqrt\frac{1-p}{\mu_K}\\ \end{aligned} $$

Arbitrary interpulse distributions where the oscillator architecture implements discrete time are accurately described by the discrete-time renewal process (Muntner 1971; van Noortwijk 2008).

## Independent (non-triggered) versus triggered generators

The case where the generator is non-triggered, unrelated to the stimulus, concerns situations when the generator works continuously and pulses are accumulated only during the time window of the stimulus. The triggered case is more particular and only concerns periodic (i.e., not memoryless) oscillators: the beginning of the stimulus triggers the generator. In biology, such condition may be related to the mechanisms of attention, awareness (Steinmetz et al. 2000), and expectation, when a neural circuit of the generator or the accumulator is synchronized to stimulus events, or it is started in some circumstances, and is otherwise idle. In engineering, a clock circuit may be switched on in reaction to some event, and may be otherwise inactive to save energy.

In this section, two specific scenarios are studied where the generator starts generating pulses based on some information regarding stimulus appearance. The generator starts working when the stimulus appears at time = 0,

- just before the accumulation of pulses begins (generator start time $=-\varepsilon_1$), or
- just after the accumulation of pulses begins (generator start time $=+\varepsilon_2$).

**Fig. 9:**
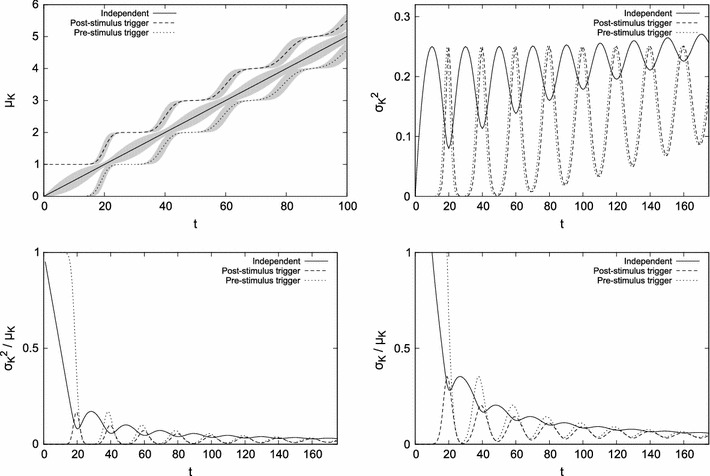
Non-triggered and triggered generators; means μ_*K*_, variances σ_*K*_^2^ and their ratios are shown for a normally distributed interpulse time (μ_*D*_ = 20, σ_*D*_^2^ = 4, *c* = 0.01). The* top right plot* presents variances of *K*, which are also shown as gray areas in the *top left plot*. The* bottom plots* show the variance-to-mean ratio and the coefficient of variation

Fig. 9 summarizes characteristics of these two scenarios (with $\varepsilon_1=1$ and $\varepsilon_2\to 0^+$) and compares them to the oscillator independent from the stimulus. The dependency of the clock start time on the stimulus appearance results in a specific variation of the mean in the beginning phase (top left plot). With time means stabilize and are offset to the non-biased mean by a constant factor that depends on *c* and on the shift $\varepsilon$ of the generator start time with respect to time zero.

Since the start time is fixed in both triggered scenarios, the variance of *K* is consequently lower and needs more time to stabilize (top right plot). In the beginning, there are intervals where the variance is zero due to the entirely determined behavior of the oscillator (a pulse is guaranteed to occur in some intervals and is impossible in the other intervals). This causes the extrema of variance to be shifted in phase compared to the independent generator.

The ratios plotted in the bottom panel of Fig. 9 are a consequence of lower variance and biased mean in the two triggered scenarios compared to the independent oscillator. Note that these discrepancies may cause the ratios to be higher than for the independent oscillator, and their magnitude is based on the amount of shift $\varepsilon$ of the start time with respect to time zero. Based on the simulations and the numerical analysis of formulas presented earlier (in particular Eq. 4), for non-skewed *D*, the *r* component of variance oscillates around approximately $\frac{1}{12}+\frac{5}{4} c^{1.5}$ for the first triggered scenario and around $\frac{3}{4}(c-\frac{1}{3})^2$ for the second scenario.

Should such dependence of a pacemaker and stimulus occur in biological systems (i.e., a periodic pacemaker generates the first pulse in response to the stimulus), it could make the interpretation of experimental data quite difficult; this will be further discussed in the following section.

## Relation to Weber’s law

The original Weber’s law states that the change in a stimulus’s magnitude $\Updelta s$ that will be just noticeable (Just Noticeable Difference, JND) is a constant ratio of the original stimulus *s* (Gibbon 1977; Rammsayer 2000). Therefore, the Weber fraction, $\Updelta s/s,$ should be constant. The fraction can also be interpreted as $\frac{\sigma_K}{\mu_K}:$ the standard deviation σ_*K*_ of the estimates of the stimulus divided by the magnitude of the stimulus, μ_*K*_ (Luce 1963). While the original Weber’s law has often been reported to hold for various senses including perception of time (Gescheider 1997; Grondin et al. 2001; Wearden 2003), there are many cases where it cannot adequately describe experimental data (Wearden 2008; Rammsayer 2000; Bizo et al. 2006; Lewis 2009).

**Fig. 10:**
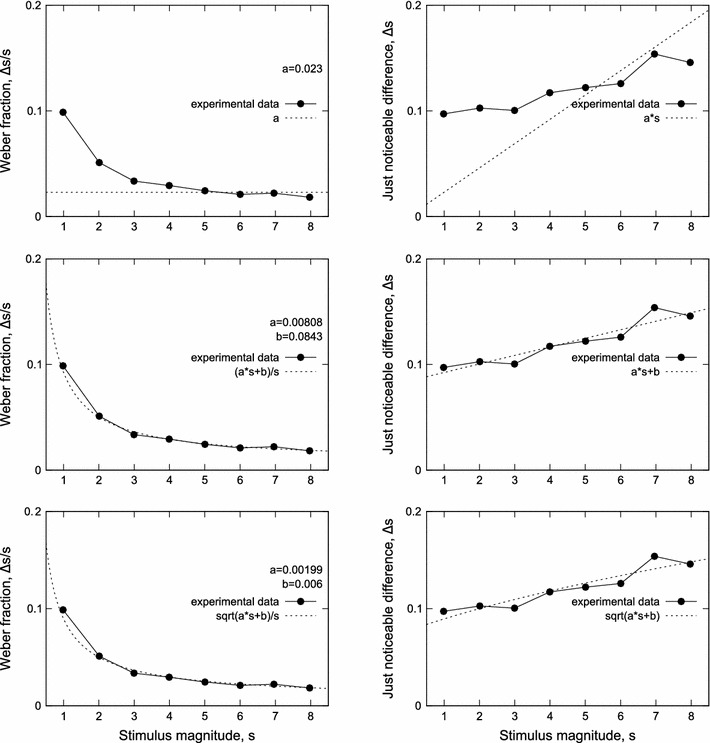
Various functions approximating experimental data. In all charts, the same dataset is shown, only regression functions differ. Stimulus magnitude *s* corresponds to μ_*K*_ from earlier sections, and $\Updelta s$ corresponds to σ_*K*_

Fig. 10 shows a typical experimental result on animals (e.g., humans discriminating whether a tone was short or long) where the Weber fraction is not constant: it varies for small and large magnitudes of the stimulus *s*. The left column shows the Weber fraction, $\Updelta s/s,$ as a function of *s*, and the right column shows $\Updelta s$ (JND) as a function of *s*.

While the constant Weber fraction quite often does not fit to experimental data as illustrated in the first row in Fig. 10, note that when the range of the stimulus magnitude is small enough and/or the precision of measurements is low enough, the Weber’s law will be reported to hold. For other cases, modified and generalized forms of the Weber’s law (second row in Fig. 10 is an example) have been proposed (Gescheider 1997; Augustin 2009; Killeen 1987) that include additional parameters (degrees of freedom), and therefore they can obviously better describe results of experiments.

For the periodic oscillators studied in this work, the variance σ_*K*_^2^ of the number of pulses grows approximately linearly with the magnitude of the stimulus μ_*K*_ so the standard deviation grows as a square root of the magnitude of the stimulus. The bottom row in Fig. 10 shows that a square relationship between the magnitude of the stimulus and its standard deviation ($\Updelta s=\sqrt{as+b}$) would fit this sample dataset as well as the generalized Weber fraction. The latter formula describes relation similar to scaled Eq. 9.

While the difference between the last two rows in Fig. 10 can be barely seen, they in fact illustrate two different laws: the scalar property where the standard deviation is proportional to the mean (Gibbon 1977; Gibbon 1992), and the non-scalar property where the variance is proportional to the mean. Without additional information, either of the two properties could be found in data. Still, the two functions differ; for growing magnitudes of *s*, the Weber fraction approaches *a* in the second row, and approaches 0 in the last row (just as Eq. 12). This difference can be hardly discovered when experimental data are scarce; see Fig. 6 and also the independent oscillator solid line in Fig. 9 bottom, left and right.

The risk of misinterpretation is also present when the precision of measurements is high, but the number of tested stimulus magnitudes and their range is small (e.g., three stimulus lengths). The apparently straight line that goes through the three points may also be a flat slope of the square root function (Fig. 7, top left). In addition, for periodic pacemakers, the influence of the regularity of the pacemaker on measured variance can be large enough to disrupt monotonicity (Fig. 9, bottom).

**Fig. 11:**
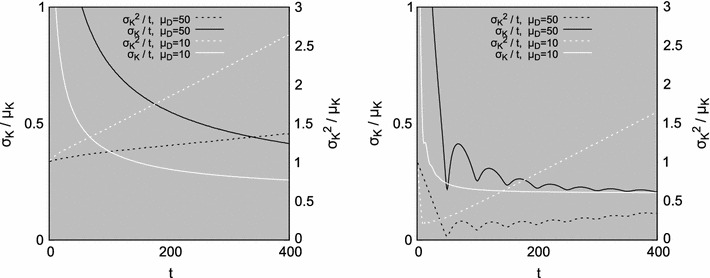
Scalar property as the effect of multiplying the observed number of pulses in each experiment by $\mathcal{N}(1,0.04),$ before σ_*K*_^2^ is calculated. Two interpulse distributions are shown, each with μ_*D*_ = 50 (*black*) and μ_*D*_ = 10 (*white*);* left*: exponential, $\lambda=\frac{1} {\mu_{D}},$ *right*: normal, σ_*D*_ = 2.* Solid line* demonstrates the coefficient of variation that exhibits the scalar property for *t* large enough, *dotted line* is the variance-to-mean ratio

This discussion demonstrates the need to explain the underlying mechanisms of the clock–counter architecture; while variants of the Weber’s law are used to describe data, they do not provide a definitive meaning of their parameters. Various hypotheses have been suggested regarding the interpretation of parameters introduced in generalizations of Weber’s law, yet it is still unknown how these constants emerge from the neural structure and how they could be measured on the neural level. To make the clock–counter models consistent with the scalar property and the Weber’s law, additional—not yet fully confirmed on the neural level—mechanisms had to be proposed (Wearden 2003; Gibbon 1992; Gibbo 1999), as illustrated in Fig. 11 for a Poissonian and a regular oscillator.

The problem with verifying the scalar property is often the problem of *scale*. In some works, the Weber fraction determined from experimental results is reported to change (not always monotonically (Getty 1975; Bizo et al. 2006; Rammsayer 2000)), while in others it is considered constant. This alone raises concerns: if the modified Weber’s law holds and the *b* coefficient is positive (second row in Fig. 10), the Weber fraction is never constant—it decreases with increasing *s*. Still, for a limited range of *s* and/or for a small *b*, it can be argued that $\Updelta s/s$ is constant. Analogously, a rapid drop (Getty 1975; Wearden 2008) in the value of $\Updelta s/s$ for small magnitudes of *s* can be reported for both functions shown in Fig. 6, and appropriate coefficients can be sought to fit both functions to experimental data. It is however hard to draw meaningful conclusions as long as these coefficients are not grounded in neurobiology, regression functions have many degrees of freedom, or experimental results are imprecise.

Actions taken to deal with the scale problem and to understand perception of stimuli magnitudes generally follow two directions:

- Gathering more experimental, accurate data that cover a wide range of magnitudes of stimuli to be able to draw more reliable conclusions regarding the analytic form of relations in the data. The problem here is that for different magnitudes, different mechanisms may be employed on a neural level, so there may be no consistency in the logic that underlies the data (Rammsayer 2000; Ulrich et al. 2006; Ivry 2008; Lewis 2009).
- Trying to understand perception mechanisms at the neural level: performing low-level physiological experiments supported by a synthetic approach (i.e., building working models of these mechanisms bottom-up while ensuring that they are consistent with the current knowledge and they fit to experimental data) (Anderson et al. 2004; Komosinski 2011).

In this context, the latter approach could be called a structural or functional regression, as opposed to numerical regression from the first group of actions. It appears to be a promising source of knowledge that can help discover origins of experimental data, not just describe them analytically.

## Conclusions

This article discussed properties of the clock–counter model with periodic generators employed as the source of regularly emitted pulses. Periodic generators have been characterized by the squared coefficient of variation as a property reflecting generator inaccuracy in producing periodic pulses. These generators have been compared to the Poisson generator; for the corresponding distributions of generator interpulse time, the probability distributions of the number of pulses *K* accumulated in time *t* have been presented along with the analytical descriptions of the measurement process.

Several interpulse distributions have been implemented and tested in simulation: continuous exponential, normal, uniform, and discrete geometric, uniform, and two-point. The normal distribution can be considered a model of other non-uniform distributions, including modified exponential ones for Poisson-like spike generation process that takes into account refractory periods in neurons.

A number of numerical experiments have been performed to illustrate the influence of generator parameters on the possible outcomes of the measurement. Particular attention has been paid to the relations between measurement accuracy, measurement variability, and the mean number of pulses observed. For all the considered pulse generators (normally and uniformly distributed, as well as memoryless, and their discrete counterparts), the variance of the number of accumulated pulses σ_*K*_^2^ depends approximately linearly on time *t* and, consequently, on the expected number of pulses, μ_*K*_. This relationship, assuming correspondence of the minimal perceptible difference and the standard deviation of measurements, does not follow the Weber-Fechner law.

Finally, a few issues have been examined that concern realizations of periodic generators. The influence of the discrete generator time has been touched upon, and the scenarios of the generator being triggered by the stimulus have been analyzed and compared to the independent generator. In all these scenarios, variance of the number of pulses emitted during stimulus presentation exhibits complex, quasi-periodic behaviors. A discussion has been presented to illustrate difficulties in determining relationships between stimulus length and the mean and variance of the number of pulses when experimental data are scarce, models have many degrees of freedom, or their coefficients are not grounded in neurobiology.

Periodic generators are commonly found in nature and engineering, therefore they deserve a thorough analysis. This article concerned imperfect periodic generators employed as a part of a larger measurement architecture. With increasing amounts of data becoming available from neuroscientific experiments, these studies may not only help understand these data, but also suggest the way biological oscillators are built and used in animals to estimate magnitudes of surrounding phenomena.

## Footnotes

1 To define *F*_*K*_(*k*) for non-negative real *k*, replace *k* with $\lfloor k \rfloor$ on the right side. *F*_*K*_(*k*) = 0 for *k* < 0.
